# Evolution of ribosomal DNA-derived satellite repeat in tomato genome

**DOI:** 10.1186/1471-2229-9-42

**Published:** 2009-04-08

**Authors:** Sung-Hwan Jo, Dal-Hoe Koo, Jihyun F Kim, Cheol-Goo Hur, Sanghyeob Lee, Tae-jin Yang, Suk-Yoon Kwon, Doil Choi

**Affiliations:** 1 Plant Genome Research Center, KRIBB, Daejeon, 305-806, Korea; 2 Systems Microbiology Research Center, KRIBB, Daejeon, 305-806, Korea; 3 Department of Functional Genomics, University of Science & Technology, Daejeon 305-333, Korea; 4 Department of Plant Science and Plant Genomics and Breeding Institute, Seoul National University, Seoul, 151-742, Korea; 5 Dongbu Advanced Research Institute, Dongbu HiTek Co, Ltd Daejeon 305-708, Korea; 6 Department of Horticulture, University of Wisconsin-Madison, Madison, WI 53706, USA; 7 Omics and Integration Research Center, KRIBB, Daejeon, 305-806, Korea

## Abstract

**Background:**

Tandemly repeated DNA, also called as satellite DNA, is a common feature of eukaryotic genomes. Satellite repeats can expand and contract dramatically, which may cause genome size variation among genetically-related species. However, the origin and expansion mechanism are not clear yet and needed to be elucidated.

**Results:**

FISH analysis revealed that the satellite repeat showing homology with intergenic spacer (IGS) of rDNA present in the tomato genome. By comparing the sequences representing distinct stages in the divergence of rDNA repeat with those of canonical rDNA arrays, the molecular mechanism of the evolution of satellite repeat is described. Comprehensive sequence analysis and phylogenetic analysis demonstrated that a long terminal repeat retrotransposon was interrupted into each copy of the 18S rDNA and polymerized by recombination rather than transposition via an RNA intermediate. The repeat was expanded through doubling the number of IGS into the 25S rRNA gene, and also greatly increasing the copy number of type I subrepeat in the IGS of 25-18S rDNA by segmental duplication. Homogenization to a single type of subrepeat in the satellite repeat was achieved as the result of amplifying copy number of the type I subrepeat but eliminating neighboring sequences including the type II subrepeat and rRNA coding sequence from the array. FISH analysis revealed that the satellite repeats are commonly present in closely-related *Solanum *species, but vary in their distribution and abundance among species.

**Conclusion:**

These results represent that the dynamic satellite repeats were originated from intergenic spacer of rDNA unit in the tomato genome. This result could serve as an example towards understanding the initiation and the expansion of the satellite repeats in complex eukaryotic genome.

## Background

The large variety of genome sizes found throughout the plant kingdom is mainly attributed to species-specific differences in ploidy and repetitive DNA content [1]. Repetitive DNA can be divided into two categories: interspersed repeats, which are individual repeat units that are distributed around the genome in an apparently random fashion, and tandem repeated DNA, whose repeat units are placed next to each other in an array. Several previous studies have uncovered interspersed repeats, the retrotransposons, which are usually the most abundant form of repetitive DNA in plants with large genomes [2,3]. The transposition mechanism of these repeats has been well characterized with respect to interspersed repeats. Multigene families including ribosomal RNAs (rRNA) as well as noncoding sequences such as satellite DNA, minisatellite sequences and microsatellite sequences are often arranged in tandem arrays [4,5]. Tandemly repeated DNA is primarily found at centromeres, subtelomeric regions, and heterochromatin. Recently, a number of new satellite repeats have been described in higher plants using cytological techniques [6-8].

Ribosomal DNA is one of the most well-characterized tandem arrays and is made up of genes that are transcribed into the components of the ribosome [9]. The repeated unit consists of the 18S, 5.8S, and 25S rRNA genes, external transcribed spacers, internal transcribed spacers, and an intergenic spacer (IGS). The coding regions of rDNA are highly conserved among eukaryotic organisms, whereas the sequence of the noncoding IGS region varies broadly between even closely-related species. This observation has been explained by the model of horizontal or concerted evolution, originally proposed by Brown *et al*. [10]. The identity of coding sequences from different species can be explained to have occurred through the maintenance of sequences with strong purifying selection. However, in plants, such as legumes [11], potato [12], and tobacco [7,13], highly amplified satellite repeats with sequence homologous to the IGS subrepeats of rDNA have been reported to exist in dispersed patterns over several chromosomes. In these genomes, the IGS subrepeat-homologous satellite sequences occur in blocks independent of the rRNA gene cluster [12,14,15]. The satellite sequences described in plants often have erratic distributions and large differences in abundance, between even closely related species [7,15]. The discovery of satellite repeats homologous to the IGS of 45S rDNA induced speculation that satellite repeats might be originated from 45S rDNA. The mechanism of satellite repeat generation has been explained by several hypotheses. (1) Satellite repeats could have arisen through repeated and random unequal crossing over [16], (2) by replication slippage and unequal crossing over with subsequent expansion [17], and (3) by the products of rolling circle replication of extrachromosomal circular DNAs that became re-inserted into the genome [18,19]. The segmental duplication of large arrays of satellite repeats has also been proposed to be the primary mechanism responsible for their amplification, contributing to the rapid reshuffling of CentO satellites in rice centromere [20,21]. However, to date, there is no clear explanation on how the sequence of the rDNA unit escaped from the highly efficient concerted evolutionary mechanisms that keep it so well conserved. To better understand the origins of satellite repeats, it is necessary to find and compare sequences from genomes in different stages along the path of satellite repeat generation [22]. Here we report the discovery of a satellite repeat that is highly homologous to IGS of the 18S–25S rRNA genes in tomato. The comparison of sequences from several BAC clones containing rDNA in various stages of modification has provided a plausible explanation for how IGS homologous satellite repeats were developed from the well-conserved rDNA unit.

## Results

### Cytological localization of 45S rDNA and IGS-homologous repeats in the tomato genome

**Figure 1:**
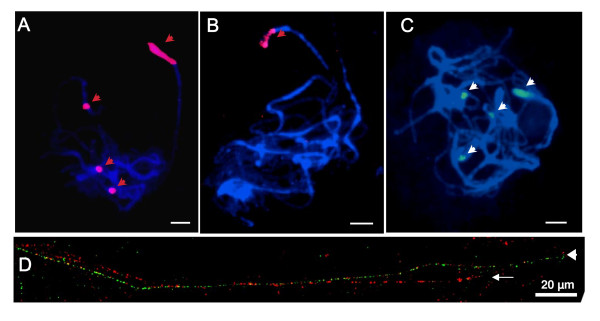
**The distribution of IGS-homologous satellite repeats**. (A) FISH signals (red) obtained with probe derived from Hba0007F24, containing tomato rDNA (red arrow head). Bar, 10 μm (B) FISH signal (red) obtained from heterologous rDNA probe, pTa71, for wheat 25-18S rDNA (red arrow head). Bar, 10 μm (C) FISH signals (green) obtained with probe, pIGS, for type I subrepeat of rDNA (arrow head). Bar, 10 μm (D) FISH signal on DNA fibers prepared from *S. lycopersicum *with pTa71 (green) and pIGS (red) probes. IGS homologous satellite repeat (arrow) and rDNA array were detected (arrow head).

As part of the international tomato genome sequencing project, we employed fluorescence *in situ *hybridization (FISH) analysis for confirmation of the position of genetic marker-anchored BAC clones on chromosome 2 [23,24]. LE-HBa0007F24, a clone anchored at genetic marker cLER-1-H17, produced very strong signals in the nucleolus organizing region (NOR) of chromosome 2 and on three other chromosomes (Figure 1A). However, hybridization with wheat 45S rDNA (pTa71, GeneBank accession number: X07841) produced only one signal focused on the short arm of tomato chromosome 2, indicating that this is the only location of rDNA repeats (Figure 1B). When the nucleotide sequences of tomato and wheat 45S rDNAs were compared, the 18S rRNA genes were 96% identical, whereas the IGS sequences had very low identity [25]. Therefore, we speculated that only the short arm of chromosome 2 contains the canonical 45S rDNA unit and that the foci on the other three chromosomes do not contain the coding sequences of 45S rDNA, but some IGS sequences.

In order to test our hypothesis, we performed FISH analysis with a tomato-specific IGS probe (pIGS) made from 483 bp of tomato sequence amplified from the type I subfamily IGS of 25-18S rDNA (Figure 1C). Like the tomato 45S rDNA probe and unlike the wheat 45S rDNA probe, the tomato type I IGS probe hybridized to loci on four chromosomes. FISH analysis on extended DNA fibers prepared from *S. lycopersicum *confirmed that there are two types of IGS organization in the tomato genome (Figure 1D). One is co-localized with the coding sequence of rRNA genes and the other is linearly stretched over a 300 kb region that lacks rRNA genes. Because FISH analysis had demonstrated that the HBa0007F24 clone is derived from the same chromosomes that hybridize to the tomato 45S rDNA probe, a probe was made from the partial 18S rDNA fragment of HBa0007F24 and tested in another FISH experiment (data not shown). This probe hybridized only to the NOR on the short arm of tomato chromosome 2. These data indicate that there is a single known canonical 45S rDNA block and three IGS homologous satellite repeats that are independent of rRNA genes in the *S. lycopersicum *genome.

### Sequence analyses reveal that transition of rDNA is initiated in the NOR

**Figure 2:**
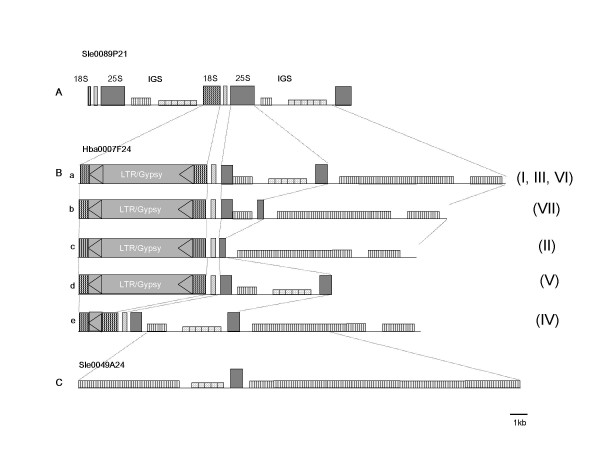
**rDNA variants in *S. lycopersicum *genome**. (A) Typical rDNA units in Sle0089P21 (18,122 bp). (B) Modified rDNA units found in BAC clone, HBa0007F24 (131,560 bp). There are seven variant repeats in the BAC clone. Retrotransposon sequences and extra-IGS regions are detected in 18S rRNA and 25S rRNA genes, respectively. Roman numerals in parentheses are the numbers of rDNA units in HBa0007F24. (C) More expanded IGS sequence in Sle0049A24 (28,040 bp).

To determine how the satellite repeats are generated from the 45S rDNA array, we determined full sequences of two BAC clones in distinct stage of divergence of rDNA repeat: HBa0007F24 (131,560 bp, GeneBank accession number: AC215351) of which end sequence was 89% identical to tomato 45S rDNA (GeneBank accession numbers: AY366528, AY366529) and Sle0089P21 (17,800 bp, GenebBank accession number: AC215459) which contains two copies of canonical rDNA (Figure 2A–B). Sequence comparison of two BAC sequences revealed dynamic changes in the HBa0007F24 sequence which consists of seven diverged truncated rDNA units with similar composition but different lengths of elements. Essentially every unit has 18S, 25S, 5.8S, and IGS. The modified 18S and 25S rDNAs showed 92–94% and 89–91% identity to typical rDNAs, respectively. Annotation of the sequence revealed that there are three significant modifications of the 45S rDNA in the HBa0007F24 clone (Figure 2). First, the 18S rDNAs were interrupted by LTR-type retrotransposons. Second, the 25S rDNAs were fragmented by the IGS-like sequences. Third, IGS sequences which have 3 to 4 times longer than normal rDNA unit were found between the 25-18S rDNAs.

To further characterize the IGS homologous repeat sequences in the tomato genome, we selected and sequenced the BAC clone, Sle0049A24 (28,040 bp), which has expanded IGS sequences at both ends, according to the results of a BLAST search of the BAC end sequence database (Figure 2C). The majority (85%, 23,858 bp) of the total 28,040 bp sequence of Sle0049A24 (GeneBank accession number: AC225927) is a long IGS stretch, which are 86% identical to subrepeat I. The remainder of the sequence (1,753 bp) is partial 25S rDNA and subrepeat II of IGS. The length of the repeated unit was well-conserved as 52–53 bp-long throughout the 21,607 bp of continuous subrepeat type I sequence.

### LTR retrotransposons inserted in the 18S rDNAs were polymerized by uneven recombination

**Figure 3:**
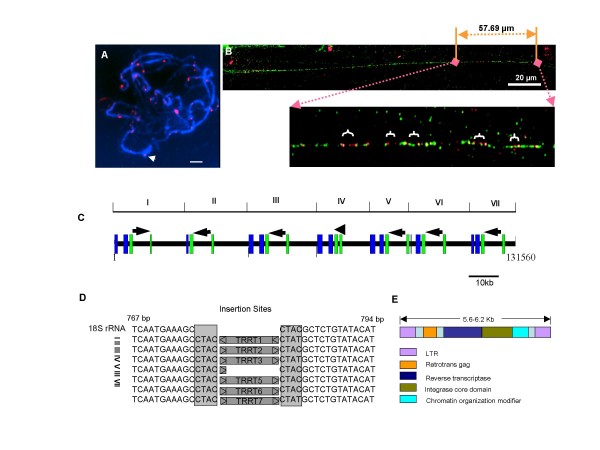
**Distribution of TRRT in the tomato genome**. (A) Chromosomal localization of TRRT (red signal). FISH analysis of pachytene stage *S. lycopersicum *was probed with TRRT. TRRT was localized on the heterochromatin of all chromosome include NOR of chromosome 2 (arrow). Bar, 10 μm (B) Fiber FISH shows that the TRRTs (red signal) are localized in the rDNA array (pTA71, green signal). (C) Arrows indicate the direction of seven TRRTs present in the HBa007F24 clone. TRRT in unit IV has solo LTR sequence. Blue box (18S rDNA) and green box (25S rDNA) indicate fragmented rRNA genes. (D) Comparison of flanking sequences of TRRTs with those of typical 18S rRNA genes. Seven TRRTs are inserted in the same site and create 4 bp TDS (shadow boxes). (E) Organization of TRRT.

Sequence comparison of HBa0007F24 and Sle0089P21 showed that the tomato rDNA related retrotransposons (TRRTs) were inserted at the same location of the seven 18S rDNAs in HBa0007F24 (Figure 3). Following the accepted system for retrotransposon nomenclature [26], it was classified as a Ty3-Gypsy like LTR retrotransposon. TRRT2 through TRRT7 are orientated in the direction of rDNA transcription, and TRRT1 has the opposite orientation (Figure 3C). To determine the reason for the opposite orientation of TRRT1, we analyzed the flanking sequences of each TRRT (Figure 3D). We found that each TRRT has the same flanking sequences (CTAC), indicating that TRRTl or others were inverted by recombination after insertion rather than inserted in the opposite orientation. Therefore, we assume that this inversion was mediated by rearrangement among duplicated segments.

**Figure 4:**
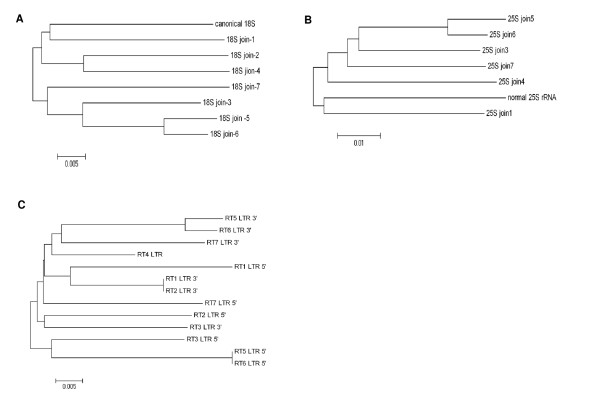
**Phylogenetic analysis of variants in HBa0007F24 sequence**. Neighbor-Joining tree obtained for 18S rDNA (A), 25S rDNA (B) and LTRs of TRRT(C). Fragmented 18S rDNA and 25S rDNA in HBa0007F24 put together deleting the inserts (18S joins, 25S joins). Comparison of phylogenetic distance of 13 LTR pairs of 7 retrotransposons shows that LTR pair of each retrotransposon is not clustered together implying that the retrotransposons was duplicated rather than transposition for LTR retrotransposon amplification. Opposite position LTRs (RT5 LTR 3': RT5 LTR 5' or RT6 LTR 3': RT6 LTR 5') of TRRT5 or TRRT6 have the most divergent sequence, but the same position LTR (RT5 LTR 3': RT6 LTR 3' or RT6 LTR 5': RT5 LTR 5') of TRRT5 and TRRT6 are closely clustered.

Phylogenetic analysis of 13 LTRs belonging to the seven TRRTs of HBa0007F24 demonstrated that segmental duplications were a major process for TRRT amplification (Figure 4C, see Additional file 1). The results from computing the proportion of nucleotide differences between each pair of LTR sequences showed that no LTR pair of a single LTR retrotransposon was clustered together implying that the retrotransposons were duplicated rather than re-inserted via a intermediate RNA. Two LTRs of TRRT1 were the most closely clustered, whereas LTRs of TRRT5 and 6 had the most diverged sequences between any two LTRs of a single LTR-retrotransposon. However, the same positions in TRRT5 and TRRT6 (RT5-5':RT6-5', RT5-3':RT6-3') were closely related, indicating they were the most recently duplicated by recombination. Phylogenetic analysis of 18S and 25S rDNAs of HBa0007F24 with typical rRNA genes shows similar results obtained in the analysis of the LTRs (Figure 4A, B).

The retrotransposon encodes four proteins, retrotrans gag, reverstranscriptase, integrase core domain, chromatin organizing modifier, and has long terminal repeats (LTR) at both ends (Figure 3E). However, TRRT4 contains solo LTR, but does not encode the gag-pol gene, indicating that some of the sequence was lost through unequal recombination [2]. The retrotransposon insertions vary in length, from 5,645 to 6,028 bp, and share 93.61% sequence identity with each other. The 18S rDNAs associated with the TRRTs have a similar degree of sequence identity to the canonical 18S rRNA gene (92–94% identity).

Because all of the retrotransposons were found at the same position, 781 bp of the 18S rDNA, with the same flanking sequences, we examined whether the TRRT transposed site-specific manner. Using the retrotransposon sequence as a query to search GeneBank (BLASTN), we identified two tomato BAC clones, C02HBa0155E05 and C06HBa0169D11, which have the same retrotransposon, but are not associated with rDNA. The flanking sequences of these retrotransposons were different from those of HBa0007F24. FISH analysis also demonstrated that the retrotransposon sequence is present on other chromosomes as various sizes of blocks supporting that the retrotransposon was not integrated in a site-specific manner (Figure 3A).

### Duplication of the IGS between 25-18S rDNA sequences into the middle of 25S rRNA

**Figure 5:**
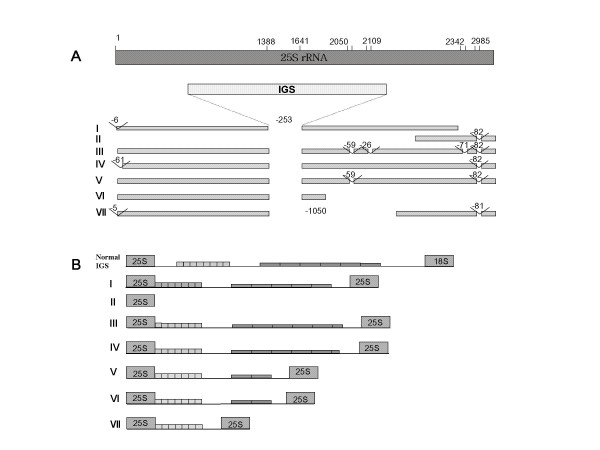
**Duplicated IGS in 25S rDNA**. (A) Deletion patterns of the 25S rDNA in the HBa007F24 clone. All IGS-insertion occurred at the same site, 1,388–1,641 bp of 25S rDNA sequence, losing 253 bp. Assuming that deletions were accumulated, the sequence of deletion events can be estimated to be IV→ V→ III from the deletion pattern. Numbers on shade box represent position of normal 25S rRNA gene. Position and length of deleted sites are indicated. (B) IGSs duplicated in 25S rDNA were compared with normal IGS of 25-18S rDNA. Two subrepeats were differently regulated: Type II subrepeats have been easily lost, but type I subrepeats were not.

Sequence comparison of HBa0007F24 and Sle0089P21 showed that IGS-like sequences are inserted at the same position of 25S rDNA, between 1,388 bp and 1,641 bp of the canonical sequence, and each interrupted copy has lost 254 bp of 25S rDNA sequence where the IGS was inserted (Figure 5A and 8). Of seven 25S rDNAs, six had IGS-like sequences in the middle of the coding region doubling the number of IGS in the 45S rDNA unit. The remaining 25S rDNA (Figure 5A-II) was missing part of its 5' end.

The size and the number of deletion of each unit of 25S rDNA demonstrated that the deletions were accumulated (Fig 5A). For example, all units (II, III, IV, V, and VII) of 25S rDNAs in the BAC clone have a deletion of 81–82 bp at the same position 2,986–3,066 bp and the deletion frequency varies from 2 to 5. Unit IV has a single 82 bp deletion, while unit III has four such deletions. Following the number of deletions in each unit, the order of recombination can be deduced as: IV → V → III. These results indicate that 25S rDNAs harboring IGS were mainly multiplied by unequal recombination from a single variant rather than individual IGSs being recombined in parallel in their respective 25S rDNA sequences.

The duplicated IGS-like sequences in the 25S rDNA commonly contain conserved sub-family repeat sequences, namely type I subrepeat, AT rich regions, and type II subrepeat (Figure 5B). The length of IGS-like sequences inserted into 25S rDNAs varies from 1,476 bp to 2,074 bp and primarily depends on the length of the type II subrepeat, but not type I subrepeat. These data indicate that type I and type II subrepeats have been differentially regulated during molecular evolution.

### Differential amplification between subfamily repeats in the rDNA intergenic spacer

**Figure 6:**
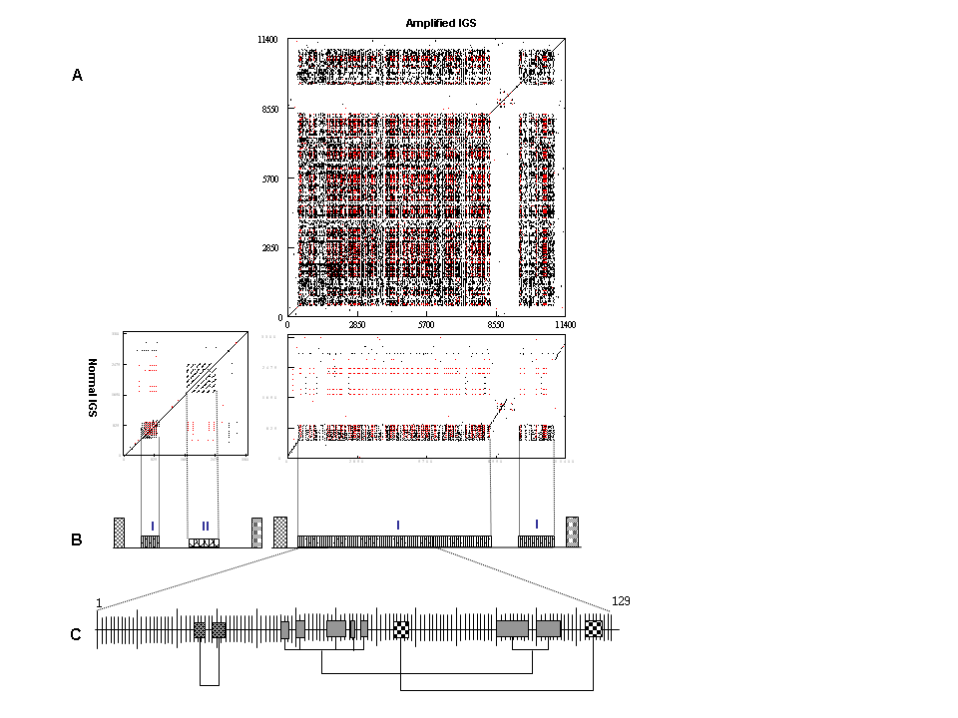
**Comparison of typical IGS and modified IGS**. (A) Dot blot analysis of normal IGS from Sle0089P21 and amplified IGS from unit IV of HBa0007F24. Red dot represents complimentary match between two sequences. Dot blot parameter: window = 15, mismatch = 0. (B) Diagram representing the structures of normal IGS and amplified IGS of 25-18S rDNA. Type II subrepeat in the modified IGS is replaced with type I subrepeat. Left-side box represents 25S rDNA and right-side box represents 18S rDNA. (C) Fragmental duplication revealed by the Neighbor-joining tree among 129 monomers of IGS unit IV. The closely related pairs of monomers are connected.

Sequence comparison of HBa0007F24 and Sle0089P21 showed that the length of the IGSs of 25-18S rDNAs (8,400 – 11,408 bp) in HBa0007F24 was 3 to 4 times longer than the normal IGS sequences (3,395 bp) present in tomato genome (Figure 6A, B). In addition, as shown in Figure 6, the type II subrepeat present at the downstream of the transcription initiation site (TIS) has been replaced by the type I subrepeat. In the amplified IGS, the type I subrepeat in the upstream of the TIS was 10–17 times longer (4,771–7,921 bp) than conventional type I subrepeat (448 bp). Furthermore, another type I subrepeat found downstream of the TIS was 2–3 times longer (1,040–1,478 bp) than the conventional type I subrepeat (448 bp). Therefore, these results strongly indicated that duplication of the IGS into the 25S rDNA occurred before the type II subrepeat replacement by the type I subrepeat, and also before the type I subrepeat amplification of the IGS located between the 25S and 18S rDNAs. Even though the length of the type I subrepeat was expanded, the monomer length was well conserved as 53 bp.

To figure out the molecular mechanisms of the repeat proliferation, we performed phylogenetic analysis of 129 repeat monomers that were identified in expanded IGS in the unit IV of HBa0007F24 sequence (Figure 6C). By analyzing the most related monomers revealed by the Neighbor-Joining tree obtained (see Additional file 2), we identified 25 pairs of monomers that are arranged in four duplicated clusters of monomers. The data obtained demonstrated that segmental duplication was occurred among the amplified type I subrepeat.

### Distribution of IGS-homologous repeats among closely-related tomato species

**Figure 7:**
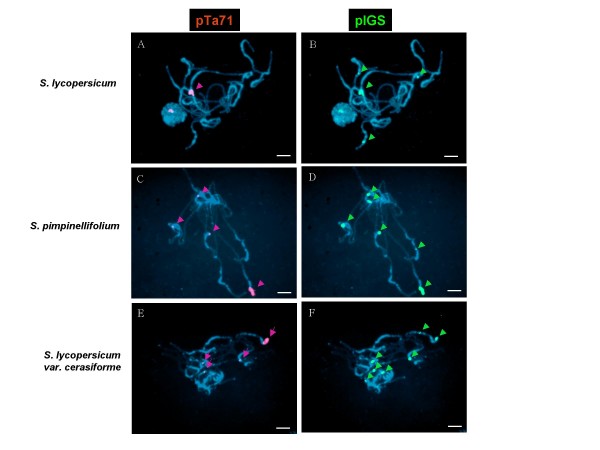
**Distribution of 45S rDNA and IGS homologous satellite repeat in tomatoes**. The number of foci is variable even among closely related species. (A) *S. lycopersicum *with pTa71 probe; (B) *S. lycopersicum *with pIGS probe; (C) *S. pimpinellifolium *with pTa71 probe; (D) *S. pimpinellifolium *with pIGS probe; (E) *S. lycopersicum *var.*cerasiforme *with pTa71 probe; (F) *S. lycopersicum *var.*cerasiforme *with pIGS probe. Bar, 10 μm

We carried out FISH analysis to study the organization of the 45S rDNA locus and IGS-homologous repeats on the Eulycopersicon red fruited subgenera [27], including *S. lycopersicum*, *S. lycopersicum *var.*cerasiforme*, and *S. pimpinellifolium*, which are very closely-related species (Figure 7, see Additional file 3). FISH analysis was applied sequentially using the pTa71 probe for the 45S rDNA locus and pIGS probe for the IGS type I on the pachytene chromosome of *S. lycopersicum*, *S. lycopersicum *var. *cerasiforme*, and *S. pimpinellifolium*. When *S. lycopersicum *chromosomes were hybridized to pTa71, a single strong signal was detected on the short arm of chromosome 2; however, in *S. lycopersicum *var. *cerasiforme *and *S. pimpinellifolium*, four signals were detected in four separated heterochromatic regions (Figure 7A, C, E). The number of signals was in accordance with the number of signals detected with the pIGS probe in *S. lycopersicum*. The number of foci detected on the pachytene chromosomes with the pIGS probe varied, as follows: four signals in *S. lycopersicum*, seven signals in *S. lycopersicum *var.*cerasiforme*, and six signals in *S. pimpinellifolium *(Figure 7B, D, F). All foci were located in regions of the pericentromeric heterochromatin. Most of the signals corresponding to the IGS repeat were stronger and more numerous than the signals from the pTa71 probe. However, the foci detected on the short arm of chromosome 2 of *S. lycopersicum *and *S. pimpinellifolium *were of a similar intensity, whether detected as with pIGS or pTa71. Taken together, the numbers of the satellite repeat vary dramatically across closely-related species and they can divide into two groups, repeats with or without rDNA coding sequence (additional file 4).

## Discussion

FISH analysis and the sequences of three BAC clones described in this study provide a good explanation on the origin and developmental procedures involved in the evolution of IGS-homologous satellite repeats because they contain both the original form and early stages of the variants in a genome. They also allow us to compare transitional sequences that make it possible to compare with previously proposed models.

**Figure 8:**
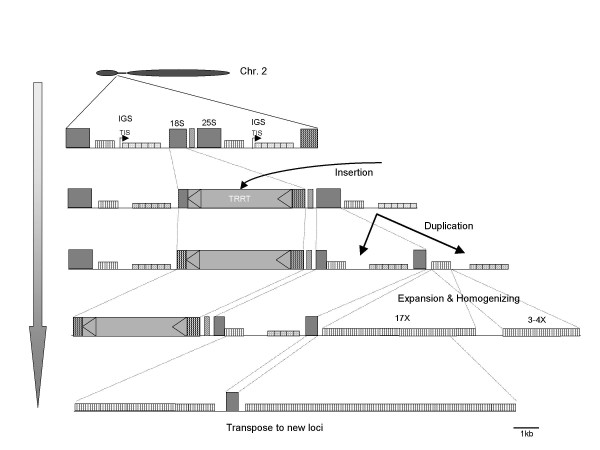
**Model for IGS homologous satellite repeat development**. Ty3-Gypsy type LTR retrotransposon (TRRT) inserted in 18S rDNA might be integrated in the array at the early stage of divergence. The TRRT inserted 18S rDNA persist and increase its copy number through recombination. Duplication of IGS into 25S rDNA seems to be occurred prior to replace type II subrepeat to type I subrepeat. Homogenized satellite repeat may be developed through amplifying type I subrepeat with segmental duplication, but removing neighboring sequences such as 25S rDNA and type II subrepeat.

Multiple mechanisms have been postulated to explain the development of satellite repeats, including unequal crossover, gene conversion, satellite transposition, illegitimate recombination, and segmental duplication [2,5,16,21,28,29]. However, the origin and the development of the early stages of satellite repeat have remained unclear because transition sequences have not been identified. Our results showed that rDNA is the origin of the satellite repeat, and repeated rearrangement and retrotransposon insertion were involved in satellite repeat initiation (Figure 8). The inserted retrotransposon in 18S rDNA might serve as sites of unequal or ectopic recombination [30]. Unequal crossover seems to be commonly employed to multiply modified rDNA units such as TRRT inserted 18S rDNAs and IGS inserted 25S rDNAs. Doubling the number of IGS into the 25S rDNA seems a very effective way of amplifying a repeated sequence. Duplication of IGS into the 25S rDNA sequence may occur prior to the amplification of the type I subrepeat of the IGS of 25-18S rDNA. Because duplicated IGS in the 25S rDNA sequence is similar with normal IGS in appearance feature while IGSs of 25-18S rDNA were highly amplified and reorganized. Segmental duplication of the repeat was also one of the major mechanisms of expanding satellite repeat [21]. The copy number of type I subrepeat in expanded IGS of 25-18S rDNA was also expanded by segmental duplication (Figure 6C)

Homogenization of the satellite repeat to single-type monomers seems to be actively conducted from the initial stage of the satellite repeat. Gene conversion and unequal crossover has been proposed as the mechanism for genome-wide homogenization of the satellite repeats [5,16,31]. Our results are consistent with the models in that both gene conversion and unequal crossover play a pivotal role in regional homogenization of the satellite repeats on initial step. Analysis of the duplicated IGS located in the 25S rDNA sequence indicated that the two subrepeats are differentially regulated in their copy number. While the type I subrepeat was conserved in copy number, the number of type II subrepeat sequence was decreased in different length between variants implying that repeated unequal crossover has occurred. Neighboring sequences of type I subrepeat such as 25S rDNA and the retrotransoposon may be deleted as a result of unequal crossover homogenizing the expanding repeat (Figure 2B). However, the replacement of the type II subrepeat to the type I subrepeat within the expanded IGS of 25-18S rDNA can be explained by gene conversion. Despite the amplification of copy number of type I subrepeats, the size of type I subrepeat monomers was relatively well conserved as 52–53 bp. It appears to be a common phenomenon for the size of satellite monomers to conserve nearly uniform within a genome [13,32,33], whereas the copy number of satellite monomers varies dramatically across species, within an organism, or on a specific chromosome between different subspecies or varieties [7,21,34].

In addition, we have postulated that satellite repeats sharing the same monomer can become separated from the original locus. The four signals detected with the pTa71 probe in *S. lycopersicum *var.*cerasiforme *and *S. pimpinellifolium *indicate that there are coding sequences of rRNA at those four loci. This situation can be explained if an rDNA block had moved to a new location outside of their original array, followed by coding region deletion and type I subrepeat amplification. However, it is more likely that the amplified IGS repeat sequence translocated outside of the rDNA array with partial coding regions attached, than that transposed sequences on multiple chromosomes independently developed satellite repeats. These assumptions are supported by our FISH results in that, with the exception of foci on the short arm of chromosome 2, very weak or no signal was detected with a partial 18S rDNA probe in *S. lycopersicum *var. *cerasiforme *and *S. pimpinellifolium*. Of course, rDNA coding sequence could be effectively eliminated in the satellite repeat of *S. lycopersicum*. In *S. lycopersicum *var.*cerasiforme *and *S. pimpinellifolium*, but not yet in *S. lycopersicum*, additional IGS-homologous satellite repeats may have been made from the original three IGS homologous satellite repeats lacking rRNA coding sequence.

Tandem repeat segments could be moved by recombinational excision of looped-out modified rDNA segments during meiotic unequal alignment of the rDNA repeating units and might re-integrate into a new location [17,28]. Indeed, this mechanism of recombinational deletion of amplified repeats was discovered in the fourth unit of rDNA in the clone, HBa0007F24. Of course, unequal recombination or illegitimate recombination could also lead to the translocation of satellite repeats [16,35].

Although the integration of mobile elements in rDNA loci has been reported in animal taxa, new insertions are rapidly eliminated from the rDNA locus by unequal crossover between sister-chromatids [35,36] and that new insertions are subject to random crossovers. Strong selective pressure against inactive rDNA units eliminates these insertions from the loci [37,38]. The LTR retrotransposon in plant genome appears to have adapted differently to survive in the rDNA locus. When the TRRT randomly inserted into the 18S rRNA gene, unequal recombination might have occurred to make the array uniform. However, instead of removing the TRRT from the rDNA, it increased the number of genes modified with the insert. Evidence of the effort to remove the TRRT is also present in the sequence: Solo LTR unaccompanied by the rest of the transposon genes may be a remnant of a retrotransposon that has been removed by unequal recombination [3]. Similar constitution of sequence containing a rDNA variant and retrotransposons was found on the heterochromatin of *Brassica rapa*. [39]. In addition, amplified satellite repeats with sequence homologous to the IGS subrepeat of rDNA have been reported in several plants [12-15], although the preferential amplification or elimination of a repeat is highly variable across species. For example, the 2D8 satellite repeat found in potatoes consists of ~3 kb monomers of AT-rich and GC-rich subrepeat clusters, showing high sequence similarity with type I and AT-rich regions of IGS in rDNA [12]. The A1/A2 satellite repeat of tobacco has sequence similarity to regions downstream of the TIS of IGS [7].

## Conclusion

We identified IGS-derived satellite repeats in tomato genome. By analyses of multiple transitional sequences, we clearly showed the origin and the growing procedure of the satellite repeat in tomato genome. Our results also suggested the molecular mechanisms of proliferation and homogenization of the satellite repeat in tomato genome by showing multiplication procedure of modified rDNA units and amplification/deletion of different subrepeats within the 45S rDNA.

## Methods

### Plant materials

Tomato plants (*S. lycopersicum *(LA3911), *S. lycopersicum *var. *cerasiforme *(LA4352), and *S. pimpinellifolium *(LA0417)) were grown in a controlled environment room at 26°C with 16 h light/8 h dark cycles.

### DNA probes

All BAC clones used for FISH mapping were identified by screening a Heinz 608 BAC library, kindly provided by Drs. S. Tanksley and J. Giovannoni at Cornell University, Ithaca, NY, USA. BAC probes were labeled with digoxigenin-11-dUTP or biotin-16-dUTP by nick translation, according to the manufacture's protocol (Roche, Switzerland). Other DNA probes used for FISH analysis were: an rDNA probe, pTa71, containing the coding sequences for the 18S–25S rRNA genes from wheat [25], pIGS, TRRT, partial 18S rRNA. Probes were amplified from the clone, HBa0007F24 with the following primer pairs: partial 18S rRNA primer (F-AATGATCCTTCCGCAG GTTTACCT, R-GCTCTGTATACATTAGCATGGGATA), 25S rRNA primer (F-CGCCCTCCTACTCTTCGGGGCCTGG, R-CAGGTTAGGCGGCATTACCCGCTGA), Retrotransposon primer (F-AATGTA TACAGAGCATAGTGTGATGTCC, R-AGTGCTCAAAGAAAGCCTACTGTCACGG), and pIGS primer (F-CGACGTACCATTT GTGCTT, R-TTACCTATGGGCAGCACACATGGTC). PCR products were cloned using pGEM-T easy Vector system (Promega, Madison, WI) and sequenced.

### Fluorescence *in situ *hybridization (FISH)

The FISH procedure applied to both mitotic and meiotic chromosomes was the same as previously reported by Koo et al. [40]. In brief, chromosomal DNA on the slides was denatured with 70% formamide at 70°C for 2.5 min, followed by dehydration in a 70%, 85%, 95%, and 100% ethanol series at -20°C for 3 min each. The probe mixture, containing 50% formamide (v/v), 10% dextran sulfate (w/v), 5 ng/μl salmon sperm DNA and 500 ng/μl of labeled probe DNA, was heated at 90°C for 10 min then kept on ice for 5 min. Twenty microliters of this mixture was applied to the denatured chromosomal DNA and covered with a glass cover-slip. Slides were then placed in a humid chamber at 37°C for 18 h. Probes were detected with avidin-FITC and anti-digoxigenin Cy3 (Roche, Switzerland). Chromosomes were counterstained with 1 μg/μl DAPI (Sigma). The signals were detected with a Cooled CCD Camera (CoolSNAP, Photometrics, Pleasanton, CA). Images were processed with software (Meta Imaging Series™ 4.6) using Leica epi-fluorescence microscope equipped with FITC-DAPI two-way or FITC-rhodamine-DAPI three-way filter sets (Leica, Japan). The final printed images were prepared with Adobe Photoshop 7.0.

### Fiber-FISH

Leaf nuclei were prepared, as described by Jackson *et al*. [41]. A suspension of nuclei was deposited at one end of a poly-L-lysine coated slide and permitted to air dry for 10 min. STE lysis buffer (8 μl) was pipetted on top of the nuclei and the slide incubated at room temperature for 4 min. A clean cover-slip was used to slowly drag the contents along the slide. The preparation was air dried, fixed in ethanol:glacial acetic acid (3:1) for 2 min, and baked at 60°C for 30 min. The DNA fiber preparation was incubated with a probe mixture, covered with a 22 mm × 40 mm cover-slip, and sealed with rubber cement. The slide was placed in an 80°C oven in direct contact with a heated surface for 3 min, transferred to a wet chamber, which was pre-warmed in an 80°C oven for 2 min, and transferred to 37°C overnight. Post-hybridization washing stringency was the same as for FISH on chromosome spreads. Signal detection was performed according to Koo et al. [40].

### BAC sequencing and sequence assembly

A shotgun sequencing library was constructed in the pUC118 vector for average insert sizes of 3–5 kb. BigDye Terminator chemistry v3.1 (ABI, Foster City, CA) was used for the sequencing reactions. The sequences were analyzed using an ABI3730XL automatic DNA sequencer (ABI, Foster City, CA). All of the initial sequence data obtained were analyzed with the Phred/Phrap/Consed processing [42]. Base-calling and assembling for the individual sequences were conducted through the Phred/Phrap software. The value of the Phred scores of the sequences was 30 or higher. The completely assembled sequence was edited using Consed. Sequence editing for consensus contig formation was generated by visual confirmation, using the Sequencher 4.1.5 program (Gene codes Corp., Ann Arbor, USA)

### Sequence annotation

Putative genes were primarily identified using BLASTN and BLASTX searches of GenBank . The consensus sequence of IGS monomers previously reported by Schmidt-Puchata et al. [43] was used to search against the BAC sequences, HBa007F24, SLe089P21, and Sle049A24 by BLAST. Tandem repeats were identified using Tandem Repeat Finder [44] and edited manually in DNAMAN (Lynnon Corporation, Quebec, Canada). The monomers were extracted from IGS unit IV of HBa007F24 and then aligned using ClustalX [45]. The alignments were edited manually if necessary. The Neighbor-Joining trees were built using the Kimura two-parameter method [46]. MEGA 4 was employed to calculate pairwise transition and transversion mutations [47].

## Authors' contributions

SJ, SL, JFK, DC complied results and wrote the manuscript. DK carried out all FISH analyses. SJ, CH, TY, SK carried out the bioinformatics analyses. DC directed the project. All authors read and approved the final manuscript.

## Supplementary Material

Additional file 1 **Table S1**. Pairwise distance of LTRs of TRRT. Click here for file

Additional file 2 **Figure S1**. Neighbor-Joining tree. Click here for file

Additional file 3 **Table S2**. Amplification of the IGS of 25-18S rDNA in HBa0007F24. Click here for file

Additional file 4 **Table S3**. Summary of rDNA and IGS homologous repeat sites in *Solanum *species. Click here for file
