# Supplementary material for: Evolution of ribosomal DNA-derived satellite repeat in tomato genome
Source: BMC Plant Biol. 2009 Apr 8;9:42. doi: 10.1186/1471-2229-9-42 (PMC2679016; doi:10.1186/1471-2229-9-42)
Supplement: Additional file 1 — Table S1. Pairwise distance of LTRs of TRRT. [file 1471-2229-9-42-S1.doc]

Table S1. Pairwise distance of LTRs of TRRT

|  | 1 | 2 | 3 | 4 | 5 | 6 | 7 | 8 | 9 | 10 | 11 | 12 | 13 |
| --- | --- | --- | --- | --- | --- | --- | --- | --- | --- | --- | --- | --- | --- |
| 1 |  |  |  |  |  |  |  |  |  |  |  |  |  |
| 2 | 0.048 |  |  |  |  |  |  |  |  |  |  |  |  |
| 3 | 0.065 | 0.052 |  |  |  |  |  |  |  |  |  |  |  |
| 4 | 0.048 | 0.000 | 0.052 |  |  |  |  |  |  |  |  |  |  |
| 5 | 0.065 | 0.050 | 0.056 | 0.050 |  |  |  |  |  |  |  |  |  |
| 6 | 0.067 | 0.054 | 0.054 | 0.054 | 0.060 |  |  |  |  |  |  |  |  |
| 7 | 0.052 | 0.039 | 0.048 | 0.039 | 0.050 | 0.045 |  |  |  |  |  |  |  |
| 8 | 0.076 | 0.063 | 0.067 | 0.063 | 0.058 | 0.067 | 0.058 |  |  |  |  |  |  |
| 9 | 0.069 | 0.056 | 0.060 | 0.056 | 0.067 | 0.058 | 0.048 | 0.076 |  |  |  |  |  |
| 10 | 0.076 | 0.063 | 0.067 | 0.063 | 0.058 | 0.067 | 0.058 | 0.000 | 0.076 |  |  |  |  |
| 11 | 0.069 | 0.052 | 0.058 | 0.052 | 0.067 | 0.058 | 0.048 | 0.076 | 0.013 | 0.076 |  |  |  |
| 12 | 0.063 | 0.054 | 0.063 | 0.054 | 0.060 | 0.056 | 0.045 | 0.069 | 0.063 | 0.069 | 0.063 |  |  |
| 13 | 0.065 | 0.052 | 0.056 | 0.052 | 0.063 | 0.058 | 0.043 | 0.067 | 0.056 | 0.067 | 0.056 | 0.063 |  |

1, TRRT1 LTR-5’; 2, TRRT1 LTR-3’; 3, TRRT2 LTR-5’;4, TRRT2 LTR-3’; 5, TRRT3 LTR-5’;6, TRRT3 LTR-3’; 7,TRRT4 LTR; 8, TRRT5 LTR-5’;9, TRRT5 LTR-3’; 10, TRRT6 LTR-5’;11, TRRT6 LTR-3’; 12, TRRT7 LTR-5’;13, TRRT7 LTR-3’

- Shadow boxes indicate distance of pair of each TRRT

- Distance of both ends of LTR from TRRT5 and 6 is underlined.
