# Supplementary figures and images for: Evolution of ribosomal DNA-derived satellite repeat in tomato genome
Source: BMC Plant Biol. 2009 Apr 8;9:42. doi: 10.1186/1471-2229-9-42 (PMC2679016; doi:10.1186/1471-2229-9-42)

## Slide 1
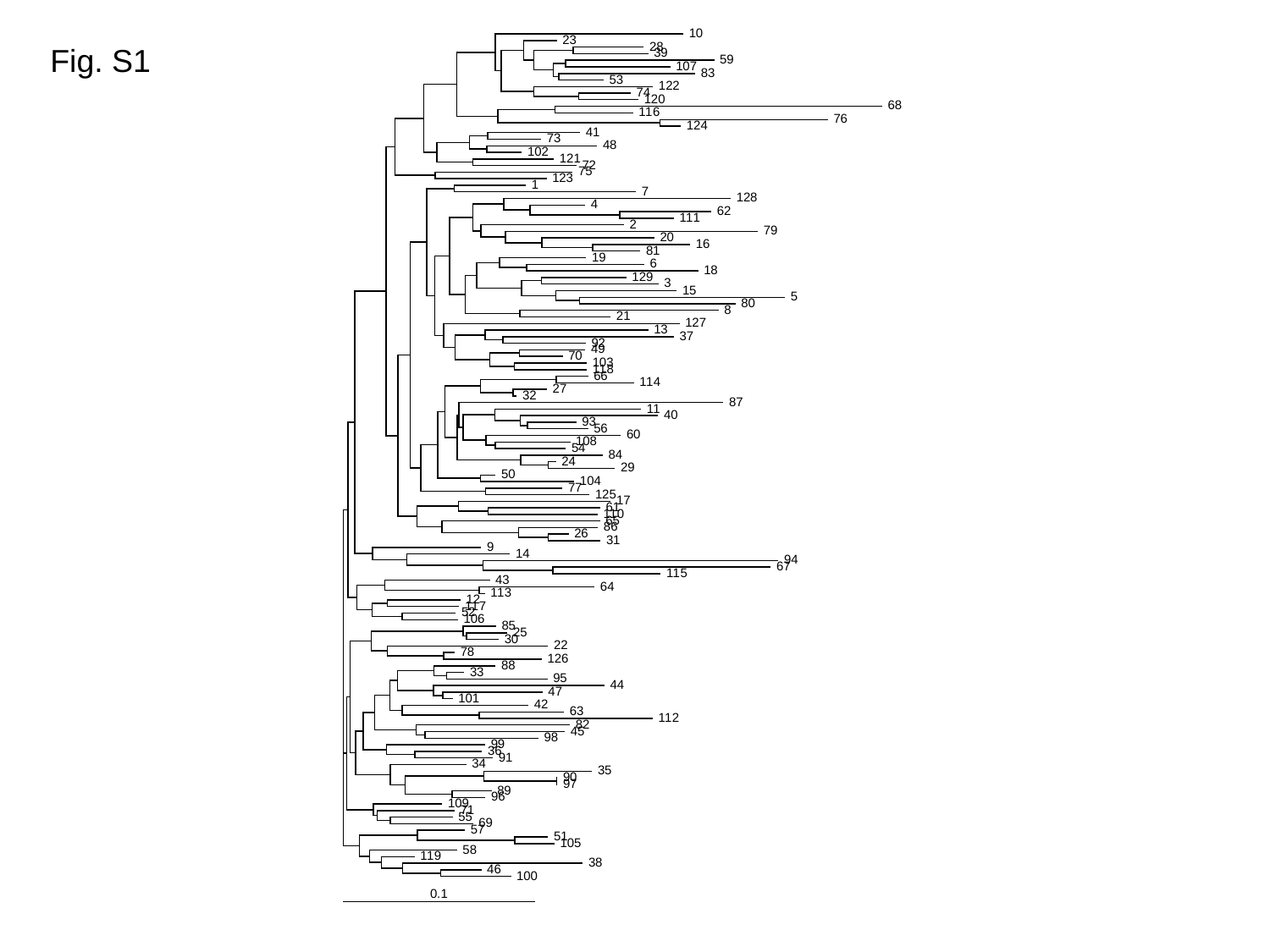

Fig. S1

Supplement: Additional file 2 — Figure S1. Neighbor-Joining tree. [file 1471-2229-9-42-S2.ppt]
