# Supplementary material for: Evolution of ribosomal DNA-derived satellite repeat in tomato genome
Source: BMC Plant Biol. 2009 Apr 8;9:42. doi: 10.1186/1471-2229-9-42 (PMC2679016; doi:10.1186/1471-2229-9-42)
Supplement: Additional file 3 — Table S2. Amplification of the IGS of 25-18S rDNA in HBa0007F24. [file 1471-2229-9-42-S3.doc]

Table S2. Amplification of the IGS of 25-18S rDNA in HBa0007F24

| IGS | Total bases  (bp) | Up stream  Type I subrepeat | AT-rich region | Down stream  Type I subrepeat^1^ | Others^2^ |
| --- | --- | --- | --- | --- | --- |
| Normal | 3253  (1X) | 448  (1X) | 723  (1X) | 846* | 853 |
| Unit 1  (I) | 11400  (3.5X) | 7921  (17.7X) | 1213  (1.6X) | 1478  (3.3X) | 146 |
| Unit 2  (II) | 10458  (3.2X) | 7796  (17.4X) | 1164  (1.6X) | 1040  (2.3X) | - |
| Unit 3  (III) | 9373  (2.9X) | 5870  (13.1X) | 1157  (1.6X) | 1425  (3.2X) | 608 |
| Unit 4  (IV) | 9204  (2.8X) | 4771  (10.6X) | 1934  (2.6X) | 1437  (3.2X) | 625 |
| Unit 5  (V) | 618 | - | - | - | - |
| Unit 6  (VI) | 8400  (2.6X) | 4756  (10.6X) | 1168  (1.6X) | 1424  (3.2X) | 613 |

* indicates the length of type II subrepeats in normal IGS

- indicates absent sequence

^1^ Type II subrepeat was replaced by type I subrepeat in HBa0007F24

^2^ indicates region from down stream type I subrepeat to 25S rDNA
