# Supplementary material for: Evolution of ribosomal DNA-derived satellite repeat in tomato genome
Source: BMC Plant Biol. 2009 Apr 8;9:42. doi: 10.1186/1471-2229-9-42 (PMC2679016; doi:10.1186/1471-2229-9-42)
Supplement: Additional file 4 — Table S3. Summary of rDNA and IGS homologous repeat sites in Solanum species. [file 1471-2229-9-42-S4.doc]

Table S3. Summary of rDNA and IGS homologous repeat sites in *Solanum* species

| Species | n | No. of chr.  with pIGS foci | No. of chr.  with 45S rDNA foci | Accession  number |
| --- | --- | --- | --- | --- |
| *S. lycopersicum*  *S. pimpinellifolium*  *S. lycopersicum*  var*. cerasiforme* | 12  12  12 | 4  6  7 | 1  4  4 | LA3911  LA0417  LA4352 |
